# Characterization on Mode-I/II Interlaminar Strength and Fracture Toughness of Co-Cured Fiber–Metal Laminates

**DOI:** 10.3390/polym17212937

**Published:** 2025-11-02

**Authors:** Mingjie Wang, Hongyi Hao, Qinghao Liu, Xinyue Miao, Ziye Lai, Tianqi Yuan, Guohua Zhu, Zhen Wang

**Affiliations:** 1 School of Automobile, Chang’an University, Xi’an 710064, China; 2 Chang’an Dublin International College of Transportation, Chang’an University, Xi’an 710018, China; 3 Department of Mechanical and Aerospace Engineering, The Hong Kong University of Science and Technology, Clear Water Bay, Hong Kong 999077, China; 4 School of Mechanical and Aerospace Engineering, Nanyang Technological University, Singapore 639798, Singapore

**Keywords:** fiber–metal laminates, interlaminar strength, interlaminar fracture toughness, failure morphology, mechanical property

## Abstract

This study systematically evaluates the mode-I (opening) and mode-II (shearing) interlaminar strength and fracture toughness of four co-cured fiber–metal laminates (FMLs): AL–CF (aluminum–carbon fiber fabric), AL–GF (aluminum–glass fiber fabric), AL–HC (aluminum–carbon/glass hybrid fabric), and AL–HG (aluminum–glass/carbon hybrid fabric). Epoxy adhesive films were interleaved between metal and composite plies to enhance interfacial bonding. Mode-I interlaminar tensile strength (ILTS) and mode-II interlaminar shear strength (ILSS) were measured using curved beam and short beam tests, respectively, while mode-I and mode-II fracture toughness ($G_{Ic}$ and $G_{IIc}$) were obtained from double cantilever beam (DCB) and end-notched flexure (ENF) tests. Across laminates, interlaminar tensile strength (ILTS) values lie in a narrow band of 31.6–31.8 MPa and interlaminar shear strength (ILSS) values in 41.0–41.9 MPa. The mode-I initiation ($G_{Ic,init}$) and propagation ($G_{Ic,\;prop}$) toughnesses are 0.44–0.56 kJ/m^2^ and 0.54–0.64 kJ/m^2^, respectively, and the mode-II toughness ($G_{IIc}$) is 0.65–0.79 kJ/m^2^. Scanning electron microscopy reveals that interlaminar failure localizes predominantly at the metal–adhesive interface, displaying river-line features under mode-I and hackle patterns under mode-II, whereas the adhesive–composite interface remains intact. Collectively, the results indicate that, under the present processing and test conditions, interlaminar strength and toughness are governed by the metal–adhesive interface rather than the composite reinforcement type, providing a consistent strength–toughness baseline for model calibration and interfacial design.

## 1. Introduction

Driven by the demand for lightweight and high-reliability structures in next-generation spacecraft and electric vehicles, lightweight alloys and fiber-reinforced polymers (FRPs) are increasingly being adopted [1,2,3]. However, each material exhibits inherent limitations under severe, multi-scenario service conditions. For instance, aluminum alloys are susceptible to localized plastic failure under concentrated impact loads although exhibiting favorable ductility [4]. FRPs offer high specific strength/stiffness and tailorability but suffer from limited low ductility and notch toughness [5,6]. To address these issues, fiber–metal laminates (FMLs), which integrate metal layers and fiber-reinforced polymer (FRP) plies through hot pressing, amalgamate the advantages of both material types [7], have been successfully applied in fuselage skins, wing structures, cargo floors, and nacelles [8,9,10,11]. One of the critical factors influencing the service performances of FMLs is the interlaminar delamination at the metal/FRP interface [12,13]. Under service conditions, interfaces of FLMs will experience both mode-I opening and mode-II shearing deformation, with crack initiation and growth strongly dependent on the mode mixity [14,15,16,17,18]. Yet, the direct comparison of interlaminar strength and fracture toughness data is often hindered by the prevalent use of a single test method, combined with significant variations in material systems, processing parameters, and experimental protocols [19]. Even when following ASTM-type standards, discrepancies in specimen preparation and data reduction introduce uncertainty, limiting extrapolation reliability [20,21]. Therefore, establishing a unified strength–toughness baseline under controlled material and processing conditions is essential. The objective of this work is to establish, under controlled material/processing conditions, a unified baseline of interlaminar strength and fracture toughness across representative modes, providing transferable parameters for model calibration and engineering design.

For both net and hybrid composite laminates with the same epoxy resin system, (e.g., CFRP, GFRP, CFRP/CFRP, or GFRP/GFRP), the underlying mechanisms of mode-I/II crack initiation and propagation have been well investigated. Interlaminar tensile and shear strengths are commonly evaluated using curved beam (ASTM D6415) and short beam (ASTM D2344) tests, which yield apparent interlaminar tensile strength (ILTS) and interlaminar shear strength (ILSS) values. It is important to acknowledge that these metrics are influenced by geometric and fixture parameters, including span, roller diameter, thickness, and bend radius, and therefore should be interpreted with careful attention to the particular test setup and failure criteria [22,23,24,25,26,27]. For fracture toughness, the double cantilever beam (DCB) and end-notched flexure (ENF) tests serve as the principal methods for determining mode-I/II fracture toughness ($G_{Ic}$ and $G_{IIc}$). Considerable efforts have been devoted to improving the data reduction accuracy of mode-I fracture toughness ($G_{Ic}$), including analytical corrections based on Timoshenko beam theory [28], crack-length-free methods to accommodate fiber bridging [29], and the compliance-based beam method (CBBM) for large-deflection conditions [30]. Further refinements incorporate fractographic analysis to differentiate initiation from propagation toughness [31]. For mode-II fracture toughness ($G_{IIc}$), Blackman et al. [32] summarized effective-crack-length and data-reduction issues that underpin ENF standardization. Wang et al. [33] compared ENF/ELS/4ENF/ONF and quantified the influence of configuration and reduction method on $G_{IIc}$. Gliszczynski et al. [34] presented an experiment–FE benchmark with VCCT/CZM validation that facilitates reproducibility and calibration; Wilk et al. [20] directly contrasted ASTM D7905 and ISO 15114, showing that standards and processing choices alter $G_{IIc}$ and comparability. Nevertheless, these established methodologies face considerably greater challenges when applied to interfaces between metal and composite materials, where crack path stability, parameter reproducibility, and cross-study comparability become significantly more difficult to ensure.

The interfacial behavior of FMLs is governed by material mismatch and geometric asymmetry, which complicate the direct application of fracture models derived from homogeneous interfaces. Differences in elastic modulus, thermal expansion, curing-induced residual stresses, and adherent asymmetry significantly influence the local mode mixity and triaxial stress state near the crack tip. In addition, adhesive thickness and surface roughness alter the interfacial contact condition, affecting the near-tip stress distribution and measured fracture energy $G_{c}$ [35,36,37]. The crack path may exhibit cohesive, adhesive, or composite intralaminar failure, leading to mode-dependent apparent toughness [38]. These factors impede consistent data interpretation in standardized tests. Although tests such as T-peel, SLJ, and DLS provide apparent strength values, results are highly sensitive to specimen configuration and stress state [39,40,41]. In DCB tests, asymmetric adherends induce mixed-mode effects under large deflections, necessitating compliance-based or crack-length-free data reduction schemes [42,43]. Similarly, ENF results are influenced by structural asymmetry and metal plasticity, requiring clear differentiation between initiation and propagation toughness with effective crack-length corrections [43,44]. Despite extensive research on FMLs, the literature shows limited consistency in materials, parameter sets, and modeling approaches. Many studies rely on single loading conditions, resulting in incomplete strength–toughness datasets and an implicit assumption of pure-mode failure, despite inherent mixed-mode behavior due to asymmetric lay-ups [45,46,47]. Furthermore, in non-alternating FML architectures, strength tests such as curved and short beam bending may induce failure at composite–composite interfaces, reflecting the weakest bond rather than the target metal–composite interface [48]. It is therefore critical to design specimens with controlled interfacial architecture and adopt rigorous fractographic validation to ensure failure occurs at the intended interface. However, cross-study comparability remains limited. Many studies employ non-alternating lay-ups, focus on a single loading mode, or use inconsistent data-reduction methods, leading to data that may reflect composite–composite interfaces rather than the target metal–adhesive bond. To address this gap, this study establishes a unified experimental baseline under consistent processing and analysis protocols, ensuring failure at the target interface and providing a complete set of interlaminar strengths and mode-I/II toughness parameters.

This study investigates the interlaminar performance of four co-cured fiber–metal laminates (FMLs) fabricated with a novel alternating metal/adhesive/composite stacking sequence. This architecture ensures a controlled crack path along the metal–adhesive interface and promotes bending stiffness balance. The laminates were systematically characterized using four ASTM-standardized tests: curved beam (D6415) for mode-I interlaminar tensile strength (ILTS), short beam (D2344) for mode-II interlaminar shear strength (ILSS), double cantilever beam (DCB, D5528) for mode-I fracture toughness ($G_{Ic}$), and end-notched flexure (ENF, D7905) for mode-II fracture toughness ($G_{IIc}$). A unified data-reduction methodology was applied throughout to ensure consistency and comparability across all measured strength and toughness parameters. Scanning electron microscopy (SEM) fractography further confirmed the failure locus and correlated the fracture morphologies with the respective loading modes. Collectively, this work establishes a reliable experimental baseline to support interfacial design and performance evaluation in FML structures.

## 2. Materials and Experimental Methods

### 2.1. Specimen Preparation

**Figure 1:**
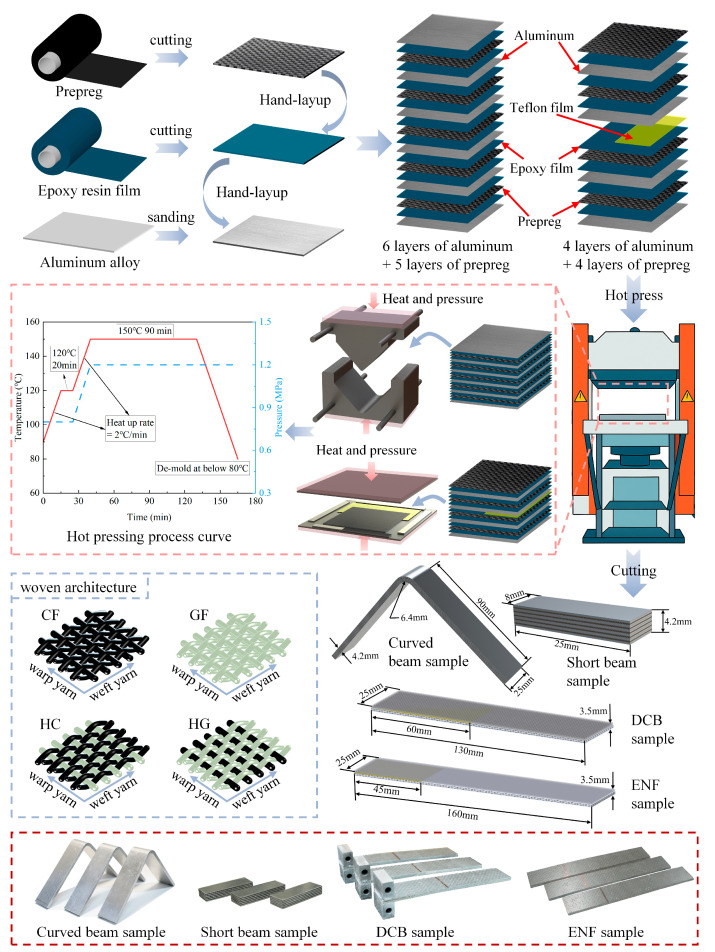
Specimen-preparation workflow and parameters for alternating metal/composite laminates: lay-up sequence, hot-press curing profile, woven architecture, and final specimen geometries with physical images (curved beam, short beam, DCB, ENF).

To ensure effective bonding between the dissimilar aluminum and FRP prepreg interfaces, an epoxy adhesive film (Provided by Yixing Ruibang Composite Materials Co., Ltd., Yixing, Jiangsu, China) was inserted at each metal–composite interface. In addition, 6061-T6 aluminum sheets with a thickness of 0.30 mm were used (Provided by Dongguan Sanshuo Metal Products Co., Ltd., Dongguan, Guangdong, China). The composite plies consisted of four types of plain-woven prepregs supplied by the same manufacturer as the adhesive film, including net CFRP (T300, plain woven fibric), net GFRP (E-glass, plain woven fibric), and two glass–carbon hybrid configurations with warp/weft arrangements of CF/GF and GF/CF. The nominal ply thickness was 0.25 mm for the prepreg and 0.20 mm for the adhesive film before pressing, and the cured thickness was slightly reduced due to resin flow and volatilization. Table 1, Table 2 and Table 3 summarized the constituent material properties of aluminum, CFRP, GFRP, and adhesive films. Prior to lay-up, aluminum surfaces were lightly abraded, while prepregs and adhesive films were thawed and cut to size. The lay-up configuration, curing cycle, fabric architecture, and specimen geometries are illustrated in Figure 1.

To mitigate spring-back, interlaminar strength panels used a symmetric lay-up of six aluminum sheets and five composite plies, whereas fracture toughness panels used four aluminum sheets and four composite plies with a polytetrafluoroethylene (PTFE) insert between the second aluminum sheet and the underlying adhesive to define the starter delamination at the interface and to approximate stiffness balance across the crack plane. For the DCB specimens, the PTFE insert length was 60 mm; for the ENF specimens, it was 45 mm. Stacks were hot-pressed in a matched mold to the specified cure profile, demolded, and water-jet cut to final geometries. Short beam (ILSS) coupons were sectioned from the cured curved beam panels to keep resin volume fraction and interfacial condition consistent within the interlaminar-strength set.

### 2.2. Experimental Methods

#### 2.2.1. Curved Beam Testing Method

**Figure 2:**
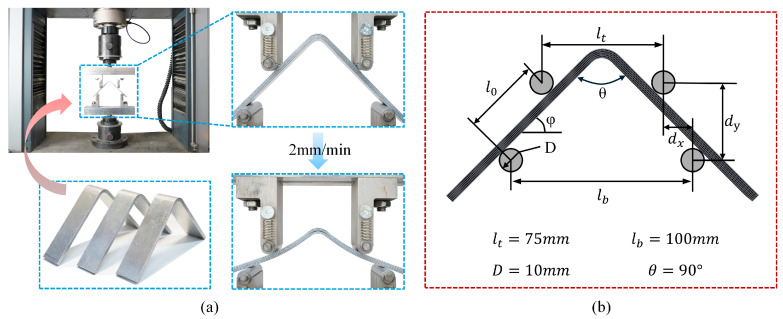
Curved beam test: (**a**) initial and post-failure states of the specimen; (**b**) schematic of fixture geometry and span dimensions.

Curved beam tests were conducted in accordance with ASTM D6415 [49] to determine the curved beam strength (CBS) and to estimate the interlaminar tensile strength (ILTS). The test setup and specimen geometry are shown in Figure 2. The coupon had an inner radius of 6.4 mm, a leg length of 90 mm, a width of 25 mm, and a nominal thickness of 4.2 mm. A four-point loading fixture was used with 10 mm diameter rollers, an upper span $l_{t}=75\;mm$, a lower span $l_{b}=100\;mm$, and a bend angle of θ=90°. Tests were carried out under displacement control with a loading rate of 2.0 mm/min and the force-displacement curve was collected simultaneously. The failure criterion was the first significant load drop or the observation of delamination in the curvature region. For each laminate type, three curved beam specimens were repeatedly evaluated, and the results are presented as the mean ± standard deviation (SD).

The curved beam strength is calculated using the load P at the first force drop as follows:

(1) $$CBS=\frac{M}{w}=\frac{P_{b}l_{0}}{w}=\left(\frac{P}{2w\cos\varphi }\right)\left(\frac{d_{x}}{\cos\varphi }+\left(D+t\right)\tan\varphi \right)$$

where φ is the angle between the loading arm and the horizontal, w is the width of the sample, t is the thickness of the sample, P denotes the total applied load (and $P_{b}$ the force carried by a single loading roller), $l_{0}$ the center-to-center distance between the upper and lower loading rollers measured along the specimen leg, $d_{x}$ the horizontal spacing between their centerlines, and D the roller diameter. For geometric and symbol placement, refer to Figure 2b. Units: P(kN), $P_{b}$(kN), M(kN·mm), w(mm), t(mm), $l_{0}$(mm), $d_{x}$(mm), D(mm), φ(°); hence, CBS has units of kN.

The interlaminar tensile strength is estimated using the approximate method given in the standard:

(2) $$\sigma_{r,max}=\frac{3\;\mathrm{CBS}}{2\;t\sqrt{r_{i}r_{o}}}$$

where $r_{i}$ and $r_{o}$ are the inner and outer radii of the curvature zone, respectively. The interlaminar strengths given here are approximate estimates; if failure does not initiate in the curvature zone, the test results are discarded.

#### 2.2.2. Short Beam Test Testing Method

**Figure 3:**
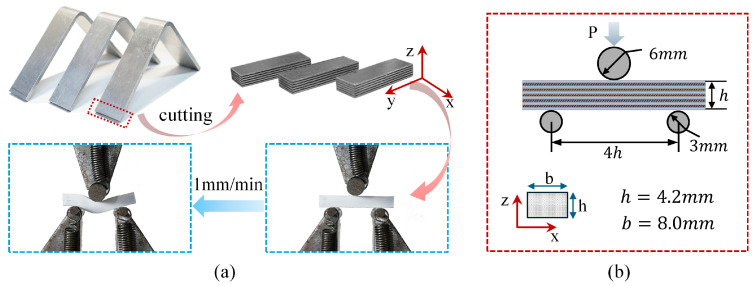
Short beam test: (**a**) specimen photographs, initial test setup, and post-test failure morphology; (**b**) fixture schematic and specimen dimensions.

Short beam tests were conducted in accordance with ASTM D2344 [50] using a three-point bending configuration (Figure 3). The loading nose and the two supports were cylindrical rollers with diameters of 6.00 mm and 3.00 mm, respectively. The span between the two lower support rollers was set to four times the measured specimen thickness (4h) and centered symmetrically. After placing and aligning the specimen on the supports, tests were performed under displacement control at 1.0 mm/min while continuously recording the load–displacement curve. The test was terminated, and the corresponding data recorded, when any of the following conditions occurred: a 30% drop in load, complete separation of the specimen into two pieces, or the loading-nose displacement exceeding the specimen thickness. For each laminate type, four short beam specimens were repeatedly evaluated, and the results are presented as the mean ± standard deviation (SD).

Data reduction followed the standard definition of the short beam strength, $F^{sbs}$:

(3) $$F^{sbs}=0.75\times \frac{P_{m}}{bh}$$

where $P_{m}$ is the maximum load observed during the test, b the specimen width, and h the thickness. It is emphasized that the internal stress state in the short beam configuration is complex; unless mid-plane interlaminar failure is clearly observed, $F^{sbs}$ should not be interpreted as the material’s “pure” shear strength.

#### 2.2.3. Double Cantilever Beam (DCB) Testing Method

**Figure 4:**
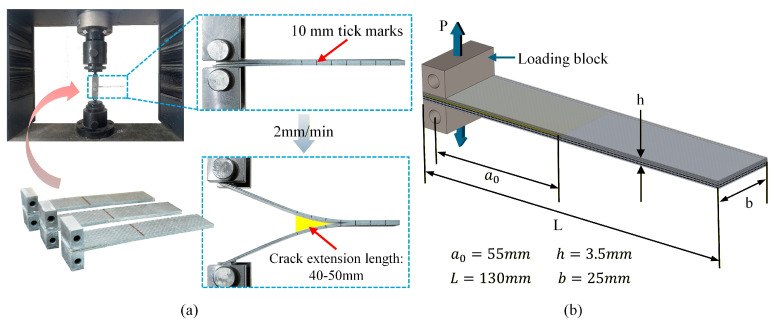
DCB test: (**a**) initial state and final failure state; (**b**) schematic diagram of fixture and specimen dimensions.

DCB tests were performed in accordance with ASTM D5528 [51]. To minimize mixed-mode effects arising from stiffness asymmetry, the laminate was a metal/composite alternately stacked hybrid (4 metal and 4 composite plies) with epoxy adhesive films at each interface; a thin release film was inserted at the mid-thickness between the central metal ply and the adjacent adhesive layer so that the upper and lower arms had comparable bending stiffness. Each DCB specimen was loaded once (monotonic, displacement-controlled); the test was stopped after the crack advanced 40–50 mm beyond the insert to minimize the influence of progressively increasing aluminum plasticity and large-deflection effects on the measured toughness. Under these conditions, the response can be regarded as an approximate pure mode-I test. For each laminate, three DCB specimens were repeatedly evaluated, and the $G_{Ic,init}$ and $G_{Ic,prop}$ results are presented as the mean ± standard deviation (SD). Figure 4 shows the fixture and specimen dimensions.

Data reduction followed the modified beam theory (MBT). First, the compliance was computed as

(4) C=δ/P

where δ is the load-point displacement and P is the applied load. A linear fit of $C^{1/3}$ versus crack length a was then used to obtain the end-correction term D (*x*-axis intercept). The mode-I energy-release rate was calculated as

(5) $$G_{I}=\frac{3P\delta }{2b\left(a+\left|D\right|\right)}$$

When large deflection was non-negligible (δ/a>0.4), a large-displacement correction factor F was applied:

(6) $$F=1-\frac{3}{10}{\left(\frac{\delta }{a}\right)}^{2}-\frac{3}{2}\left(\frac{\delta t}{a^{2}}\right)$$

(7) $$G_{I}^{*}=G_{I}\cdot F$$

where t is the load-block offset used in the test setup. The deviation-from-linearity (NL) method, taking the initiation point at the first discernible departure of the load–displacement (P–δ) curve from its initial linear segment (tangent through the origin), was used to determine $G_{I,init}$; the propagation toughness $G_{I,\;prop}$ was calculated as the average $G_{I}$ over the stable crack-growth interval.

#### 2.2.4. End-Notched Flexure (ENF) Testing Method

**Figure 5:**
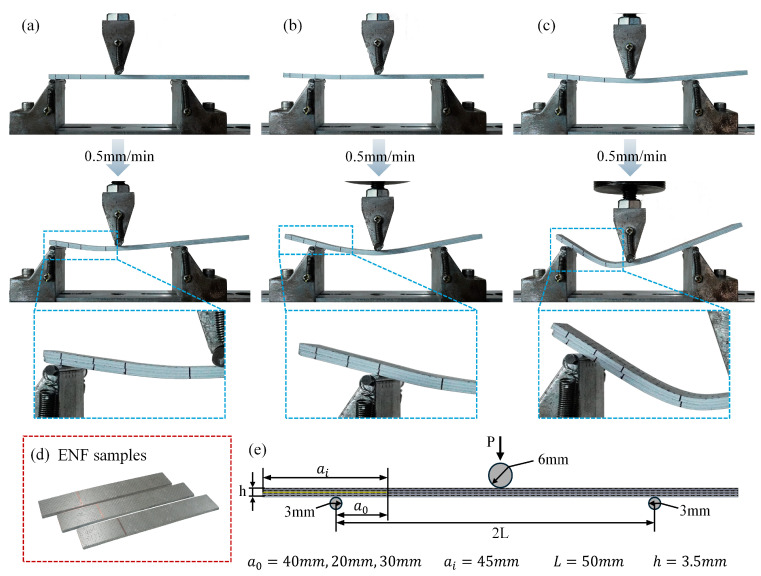
ENF test: (**a**) compliance calibration (CC) at the 40 mm placement; (**b**) CC at 20 mm; (**c**) fracture test at 30 mm; (**d**) photos of actual ENF specimens; (**e**) ENF geometry: $a_{i}=45\;mm$ denotes the insert length. The 20/30/40 mm ticks are alignment marks measured from the crack tip along the insert; when a tick coincides with the left support, the effective $a_{0}$ equals that tick value (40, 20, or 30 mm).

ENF tests were performed following ASTM D7905 [52]. The specimen surface was lightly sprayed white and reference marks were drawn at 20, 30, and 40 mm from the insert tip to define three loading positions. The support span was 100 mm and the loading nose was centered between the two supports. Tests were conducted under velocity control at 0.5 mm/min with continuous acquisition of load–displacement data. As illustrated in Figure 5, two compliance calibrations (CCs) were first carried out by placing the 40 mm and then the 20 mm marks over the crack-end support and loading until the slope of the load–displacement curve started to decrease, at which point the test was unloaded. The fracture test was subsequently performed by positioning the 30 mm mark over the same support and loading until a clear force drop or visible delamination advance occurred. For each laminate, three ENF specimens were repeatedly evaluated, and the $G_{IIc}$ results are presented as the mean ± standard deviation (SD).

For data reduction, the three load–displacement records (40 mm and 20 mm for CC; 30 mm for fracture) were processed primarily by linear least-squares fits over the initial linear regime of each curve. The inverse of the fitted slope was taken as the compliance C. Using the three crack lengths a=40, 20, 30 mm, C was regressed against $a^{3}$ to obtain the CC coefficients (intercept A and slope m):

(8) $$C=A+ma^{3}$$

To locate initiation, the load–displacement record was linearly regressed over the initial 10% of the peak displacement to obtain the initial stiffness $K_{0}$ and a reference fit $F_{fit}(\delta )$. The pointwise relative deviation

(9) $$∆F=\frac{\left|F_{means}-\left.F_{fit}\right|\right.}{F_{fit}}$$

was then evaluated; once beyond the clearly linear region, the initiation point was defined as the first position where ≥3 consecutive points exhibited ΔF>0.02–0.03, and the corresponding load and displacement were recorded. The critical point for final failure was taken as the maximum load under the 30 mm span, from which $G_{IIc}$ was computed. This continuous-deviation criterion suppresses false early nonlinearity from grip clearance or system compliance and yields a consistent initiation detection.

From the 30 mm record, the peak load $P_{max}$ was extracted and the candidate mode-II toughness was computed as

(10) $$G_{II}=\frac{3mP_{max}^{2}a_{0}^{2}}{2B}$$

where $a_{0}=30\;mm$ is the fracture-test placement and B is the specimen width.

## 3. Results and Discussion

### 3.1. Curved Beam Test

**Figure 6:**
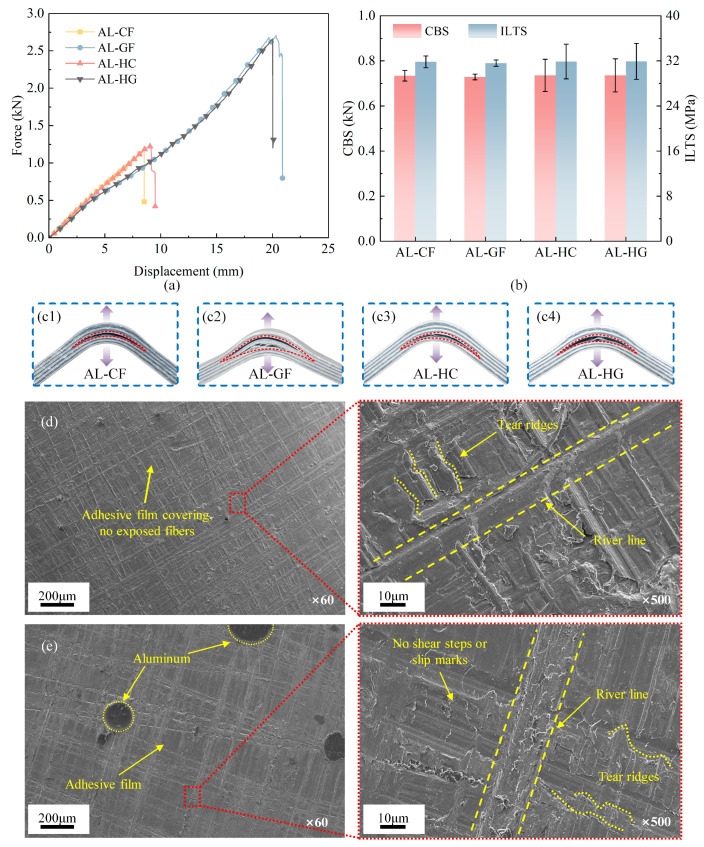
Curved beam responses with real failure photographs and microscopic failure morphologies of four FMLs: (**a**) force–displacement curves; (**b**) comparisons in curved beam strengths (CBSs) and interlaminar tensile strengths (ILTSs); (**c1**–**c4**) real failure photographs of four types of FMLs; (**d**) composite-side fracture surface; (**e**) metal-side fracture surface.

Figure 6 compares the differences in the four-point bending force, interlaminar tensile strength, and microscopic failure morphology among four FMLs. Figure 6a shows that their load–displacement curves nearly coincide at small deflections, indicating that the initial flexural stiffness is governed by FMLs geometry and the aluminum skins, with only a minor contribution from composite types. The mechanical responses diverge markedly with increasing deflection. AL–GF and AL–HG FMLs sustained stable loading to higher peak forces, demonstrating greater ductility and energy absorption, while AL–CF and AL–HC underwent unstable failure at 9–10 mm, indicating a brittle fracture signature. This difference is primarily due to the inherent ductility of the glass-dominated layers, which enhances the post-yield capacity of the FMLs. Nevertheless, the strength metrics presented in Figure 6b reveal no statistically significant differences in either the curved beam strength (CBS) or the mode-I interlaminar tensile strength (ILTS) across the four FMLs. This indicates that crack initiation under bending is largely independent of the composite type and is governed primarily by the metal–adhesive interface. Complete specimen-wise CBS and ILTS values for all laminates are given in Appendix A Table A1.

Figure 6d,e present the microscopic failure morphologies of the composite and aluminum surfaces, respectively. On the composite surface, a continuous adhesive film is observed in the low-magnification image, with no fiber exposure or breakage. High-magnification micrographs taken at the intrados of the curved beam specimens show dense tear ridges and river-line patterns oriented approximately normal to the local crack front and emanating from the inner radius; together with the absence of shear-hackle features, these observations indicate mode-I opening-dominated interfacial separation at the metal–adhesive layer under the four-point bending configuration, as shown in Figure 6d. In contrast, the aluminum surface is covered with residual resin and exhibits a matching morphology, indicating that the crack propagated cohesively within the adhesive layer near the metal–adhesive interface, rather deflecting into the composite plies. The combined evidence from SEM fractographies and mechanical responses indicates that crack initiation is controlled by the metal–adhesive interface, explaining the consistent CBS and ILTS values across four FMLs. However, the subsequent propagation of cracks and consequently the changes observed in peak load and displacement are primarily governed by the ductility and energy dissipation capabilities of the composite plies.

### 3.2. Short Beam Test

**Figure 7:**
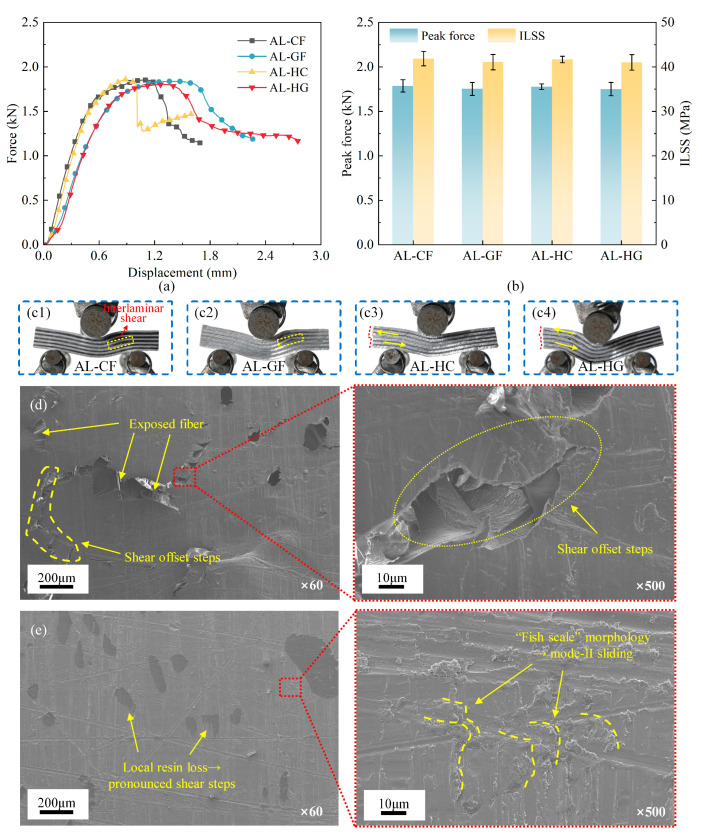
Short beam response and microscopic failure morphologies of four FMLs: (**a**) force–displacement curves; (**b**) comparisons in peak forces and interlaminar shear strengths (ILSSs); (**c1**–**c4**) real failure photographs of four types of FMLs; (**d**) composite-side fracture surface; (**e**) metal-side fracture surface.

Figure 7 presents a comparative analysis of the three-point bending force, interlaminar shear strength (ILSS), and microscopic failure morphology for the four FMLs. As shown in Figure 7a, the force–displacement responses show limited variation in ultimate strength while exhibiting slight divergence in deformability. FMLs with glass-dominated load paths (AL–GF, AL–HG) sustained loading over longer displacements and demonstrate a more gradual post-peak softening. In contrast, the carbon-dominated counterparts (AL–CF, AL–HC) are characterized by a sharper load drop after the peak, indicative of a more brittle response. Despite these differences, the peak forces clustered within a narrow band, resulting in statistically similar interlaminar shear strength (ILSS) values for all four FMLs, as shown in Figure 7b. This behavior points to a common initiation threshold and can be attributed to a limiting interlaminar strength, which dominates the short beam response irrespective of the composite type. The specimen-wise peak force and ILSS datasets are provided in Appendix A Table A2.

Figure 7d,e present the microscopic failure morphologies of the composite and aluminum surfaces, respectively. On the composite surface, low-magnification images show a displaced resin layer featuring distinct offset steps and localized resin removal that exposes underlying fibers, characteristics that are indicative of mode-II sliding rather than through-thickness opening delamination, as shown in Figure 7d. On the aluminum surface, the aluminum surface is predominantly covered by residual resin, providing additional confirmation of shear-dominated failure mechanisms. Despite being predominantly coated with resin, regions exhibiting localized detachment reveal distinct shear steps. At higher magnification, the presence of a characteristic “fish-scale” morphology offers conclusive evidence of mode-II slipping delamination, as illustrated in Figure 7e. The consistent correlation between mechanical response and microstructure confirms that short beam failure initiates by shear at the metal–adhesive interface. Consequently, the measured ILSS and peak load are largely independent of the adjacent composite type. The observed differences in the post-peak segment primarily reflect the greater strain-to-failure and energy-dissipation capacity of glass-dominated plies, which moderates softening after interfacial shear cracking.

### 3.3. Double Cantilever Beam (DCB) Test

**Figure 8:**
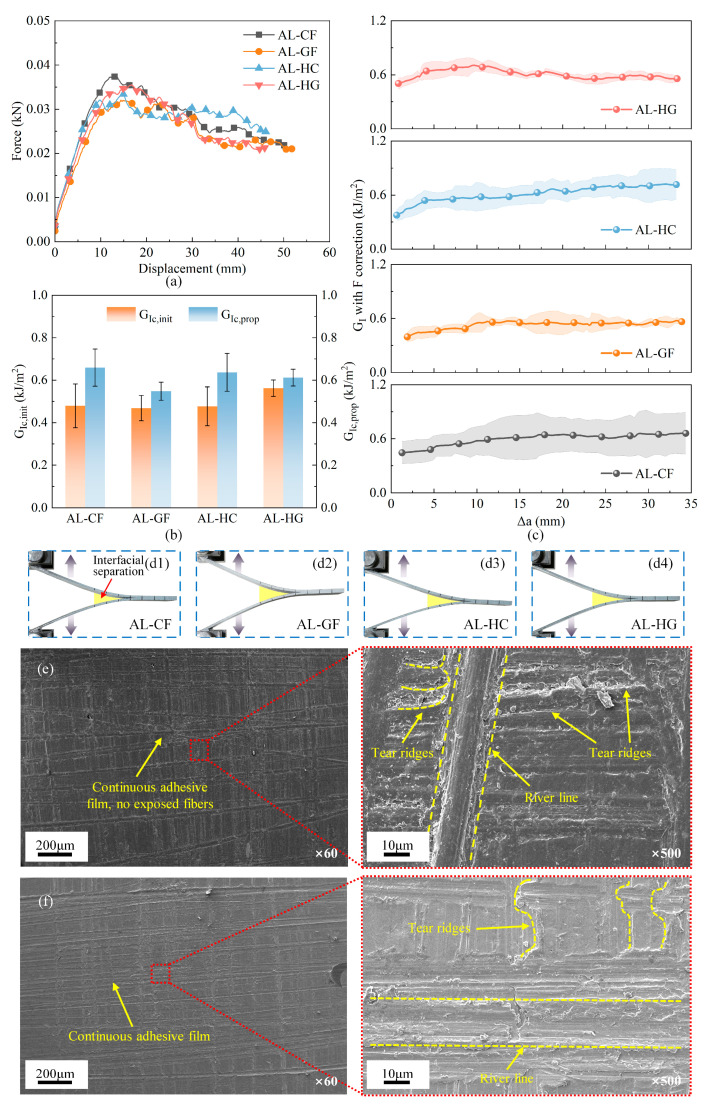
DCB response with real failure photographs and microscopic failure morphologies of four FMLs: (**a**) force–displacement curves; (**b**) comparison in initiation and propagation toughness; (**c**) $G_{I}-∆a$ histories with large-deflection F-correction; (**d1**–**d4**) real failure photographs of four types of FMLs; (**e**) composite-side fracture surface; (**f**) metal-side fracture surface.

As shown in Figure 8a–c, the DCB responses exhibit a consistent pattern across the four FMLs in all measured metrics. The load–displacement curves are characterized by nearly identical initial stiffness and comparable peak loads, followed by a gradual softening regime with minor serrations corresponding to incremental crack propagation, as shown in Figure 8a. As illustrated in Figure 8b, the F-corrected $G_{I}-∆a$ histories for all FMLs show a slight rising-R trend that quickly converges to a quasi-steady level, with inter-laminate variations being negligible and within experimental scatter. Correspondingly, the bar chart in Figure 8c shows that the initiation and propagation toughness values ($G_{IC,\;init},\;G_{IC,prop}$) for the four FMLs are statistically indistinguishable, with extensively overlapping confidence intervals and no consistent ranking order. Collectively, these findings demonstrate that both the initiation and steady-state propagation of mode-I delamination are governed by the metal–adhesive interface, showing minimal dependence on whether the adjacent composite plies are carbon-fiber- or glass-fiber-dominated. Detailed $G_{IC}$ (initiation/propagation) per specimen is summarized in Appendix A Table A3.

SEM analysis provides microstructural evidence for this material-agnostic behavior, as shown in Figure 8e,f. The composite surface is covered by a continuous adhesive film with no fiber exposure. High-magnification images of the composite-side surface (Figure 8e) show step-like tear ridges approximately normal to the crack front together with river-line patterns converging back to the initiation site; these morphologies are widely reported for mode-I opening-dominated interfacial separation in epoxy-adhesive joints. Combined with the stiffness-balanced DCB configuration and the large-deflection correction used in data reduction, these observations indicate that the present DCB fracture is opening-dominated. Similarly, the aluminum surface is predominantly coated with residual resin and displays matching opening-mode morphology, with no shear-related marks observed, as presented in Figure 8f. The observed morphologies indicate that failure transpires cohesively within the adhesive under mode-I loading, thereby restricting the crack propagation to the interfacial region. This finding substantiates that the interfacial cohesive properties, rather than the fiber type of the composite, predominantly govern the fracture toughness. Minor variations observed between different lay-ups are attributed primarily to process-induced variability at the interface and represent secondary effects.

### 3.4. End-Notched Flexure (ENF) Test

**Figure 9:**
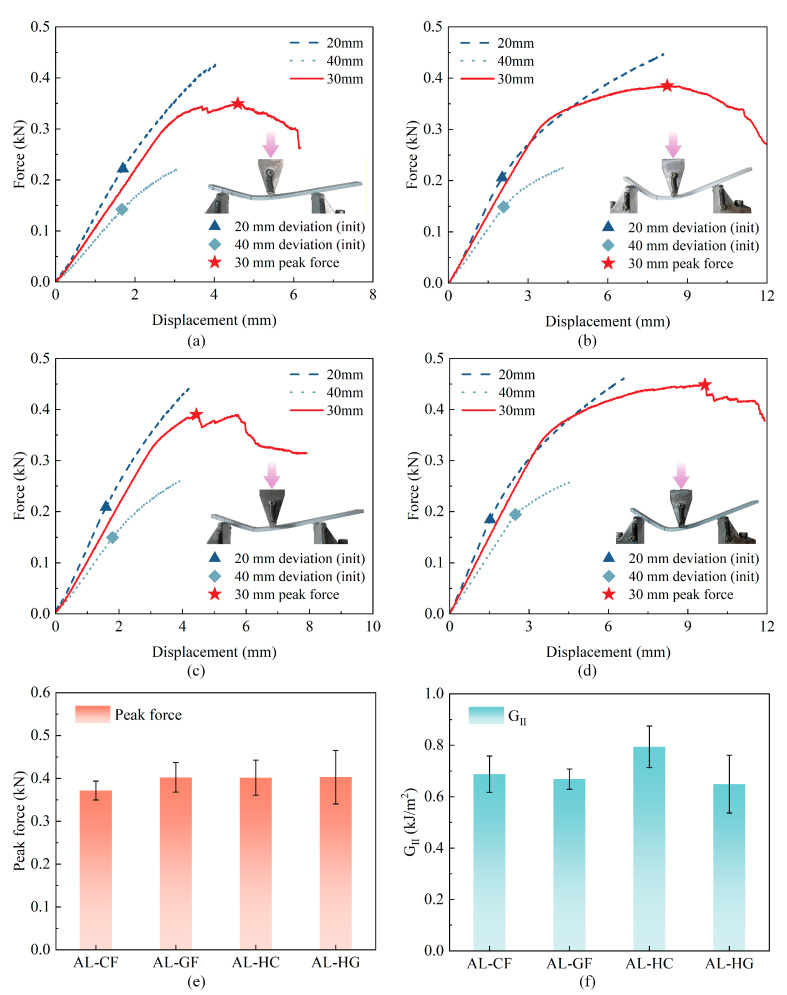
ENF testing force–displacement curves for initial crack lengths of 20/30/40 mm of (**a**) AL–CF, (**b**) AL–GF, (**c**) AL–HC, (**d**) AL–HG, where triangles and rhombus mark the first deviation from linearity (initiation) and stars denote the peak load for the 30 mm case; (**e**) comparisons in peak force at 30 mm; (**f**) mode-II fracture toughness $G_{II}$.

As shown in Figure 9a–d, the compliance decreases as the initial crack length increases from 20 mm to 40 mm for each laminate: the 20 mm configuration exhibits the steepest initial slope and the 40 mm the shallowest, as expected. However, the point of first deviation from linearity, which indicates crack initiation, does not decrease monotonically with crack length across all laminates. This initiation point occurs within a narrow and variable window. Notably, for the AL–HG laminate in Figure 9d, the deviation appears later for the 40 mm crack than for the 20 mm crack. This non-monotonic initiation behavior can be attributed to several factors, such as root rotation, localized contact and micro-friction near the crack tip, variations in adhesive thickness and surface condition, as well as the limited resolution of the linearity-deviation criterion used to detect initiation. After initiation, all load–displacement curves enter a plateau stage, eventually transitioning into a gradual softening phase with slight serrations, reflecting stable, progressive mode-II crack growth. Comparison at the 30 mm crack length reveals similar load–displacement shapes and peak forces for all laminates, as shown in Figure 9e. The resulting $G_{II}$ values in Figure 9f for AL–CF, AL–GF, and AL–HG fall within a comparable range. The slightly elevated value for AL–HC is not statistically significant, as evidenced by the overlapping error bars. Detailed $G_{IIc}$ and the fitted $C-a^{3}$ regression parameters appear in Appendix A Table A4.

**Figure 10:**
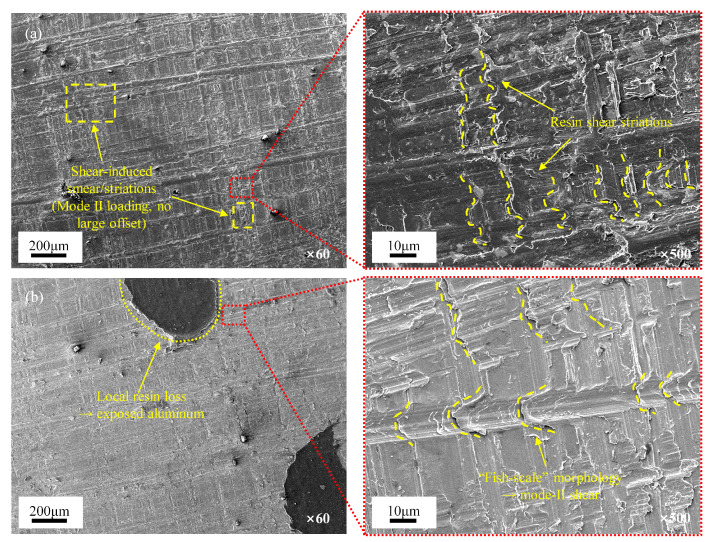
SEM fractographies of ENF specimens: (**a**) Composite side showing a continuous adhesive film with shear smearing/striations and no large offset; 500× inset highlights shear features. (**b**) Aluminum side with local resin loss exposing aluminum substrate; 500× inset shows mode-II sliding morphology.

SEM analysis of the fracture surfaces in Figure 10 provides microstructural evidence supporting the observed mechanical trends. Unlike the mode-I surfaces described in Section 3.3, the ENF fracture surfaces exhibit inclined shear-hackle and ploughing marks aligned with the sliding direction, which are characteristic of mode-II propagation. On the composite side, images at both low and high magnification reveal a continuous adhesive film with no fiber exposure or significant offset steps. Instead, the surface exhibits shear-induced resin smearing and fine striations, indicating that crack propagation occurred primarily as cohesive shear within the adhesive layer near the metal–adhesive interface, without penetrating the composite plies. On the aluminum surface, localized resin detachment reveals exposed areas of the substrate, where distinct “fish-scale” shear steps and slip marks are clearly observed, which are direct morphological indicators of mode-II sliding delamination. In contrast to the short-beam test, the ENF test introduces crack closure under bending, thereby limiting interfacial slip and fiber exposure on the composite side. However, pronounced fish-scale steps form readily due to the concentrated shear deformation on the aluminum side. The consistent mechanical and microscopic evidence support a unified mechanism where the shear properties of adhesive layer govern ENF failure, accounting for the comparable peak loads and $G_{II}$ values across laminates.

## 4. Conclusions

This study systematically characterizes the mode-I and mode-II interlaminar performance of four co-cured fiber–metal laminates (AL–CF, AL–GF, AL–HC, AL–HG) featuring an alternating metal/adhesive/composite architecture. The mode-I interlaminar tensile strength (ILTS) and mode-II interlaminar shear strength (ILSS) were evaluated by curved beam and short beam tests, while corresponding fracture toughness ($G_{Ic}$ and $G_{IIc}$) values were determined using double cantilever beam (DCB) and end-notched flexure (ENF) experiments, respectively. Fractographic analysis by scanning electron microscopy (SEM) was further conducted to identify the microscopic failure mechanisms governing interfacial separation. All reported values are mean ± SD with specimen counts per method.

(1) The interlaminar strength results show limited dispersion among AL–CF, AL–GF, AL–HC, and AL–HG, indicating no pronounced effect of reinforcement type on interlaminar strength in these FMLs. Consistently, the laminate-wise mean ILTS values (MPa, mean ± SD) are AL–CF 31.8258 ± 1.0385, AL–GF 31.8413 ± 0.3357, AL–HC 31.6447 ± 1.1674, AL–HG 31.8005 ± 3.8896; and the mean ILSS values (MPa, mean ± SD) are AL–CF 41.8652 ± 1.6321, AL–GF 41.0742 ± 1.7321, AL–HC 41.6660 ± 0.7408, AL–HG 41.0273 ± 1.7485—in line with the overall bands ILTS = 31.6–31.8 MPa and ILSS = 41.0–41.9 MPa derived from the full dataset.
(2) For fracture toughness, the DCB results show mode-I initiation ($G_{Ic,\;init}$) and propagation ($G_{Ic,\;prop}$) values (kJ·m^−2^, mean ± SD) of AL–CF 0.4789 ± 0.1030/0.6031 ± 0.1142, AL–GF 0.4438 ± 0.0597/0.5408 ± 0.0326, AL–HC 0.4765 ± 0.0915/0.6364 ± 0.0892, and AL–HG 0.5623 ± 0.0385/0.6120 ± 0.0395; the ENF results give mode-II toughness ($G_{IIc}$) (kJ·m^−2^, mean ± SD) of AL–CF 0.6877 ± 0.0707, AL–GF 0.6681 ± 0.0394, AL–HC 0.7941 ± 0.0810, AL–HG 0.6493 ± 0.1124. Collectively, $G_{Ic,\;init}$, $G_{Ic,\;prop}$, and $G_{IIc}$ values range approximately within 0.44–0.56, 0.54–0.64, and 0.65–0.79, respectively. The mode-I and mode-II toughness values for all four FMLs reside in narrow and overlapping ranges.
(3) Fractographic analysis confirms a consistent relationship between failure mechanisms and loading modes. Under mode-I loading, specimens exhibit river patterns with adhesive fibrillation, bridging, and arrest lines, along with continuous adhesive residue on the aluminum surface. Mode-II failures, in contrast, are characterized by hackle patterns, feathered steps, shear lips, and oriented rubbing marks. In all FMLs, crack propagation remained confined to the metal–adhesive interface, avoiding the adhesive–composite boundary. This consistent interfacial failure explains the insensitivity of interlaminar strength and fracture toughness to the type of composite reinforcement (carbon, glass, or hybrid fabrics).

## Figures and Tables

**Table 1:** Mechanical properties of aluminum 6061-T6.

Density	Young’s Modulus	Poisson’s Ratio	Yield Strength	Thickness
2638 kg/m^3^	70.43 GPa	0.286	117.56 MPa	0.30 mm

**Table 2:** Constituents and dimension parameters of CFRP and GFRP prepregs.

Prepreg	Reinforcement	Matrix	Fiber Type	Weave Type	Surface Density
CFRP	Carbon	Epoxy	T300	Plain woven fabric	200 g/m^2^
GFRP	Glass	Epoxy	E-glass	Plain woven fabric	300 g/m^2^

**Table 3:** Material properties of the epoxy film adhesive.

Viscosity at 70 °C	Viscosity at 80 °C	Gel Time at 120 °C	Glass Transition Temperature	Nominal Film Thickness
16,500–22,500 mPa·s	7000–9000 mPa·s	760–960 s	100 °C	0.20 mm

## Data Availability

The original contributions presented in this study are included in the article. Further inquiries can be directed to the corresponding author.

## Appendix A

Appendix A presents a comprehensive summary of the principal mechanical properties of the four types of FML configurations subjected to four distinct testing methods, including curved beam (ASTM D6415), short beam (ASTM D2344), double cantilever beam (DCB, ASTM D5528), and end-notched flexure (ENF, ASTM D7905). Table A1, Table A2, Table A3 and Table A4 list the mechanical properties. All reported data are derived from the testing of a minimum of three replicate specimens.

**Table A1:** Mechanical results of the four laminate types in the curved beam test.

Specimens No.	CBS ± SD (N)	ILTS ± SD (MPa)
AL–CF-1	757.4618	32.8443
AL–CF-2	709.5873	30.7684
AL–CF-3	734.8694	31.8647
Average	733.9728 ± 23.9498	31.8258 ± 1.0385
AL–GF-1	726.4545	31.4998
AL–GF-2	734.6027	31.8531
AL–GF-3	741.9329	32.1709
Average	734.3301 ± 7.7428	31.8413 ± 0.3357
AL–HC-1	731.1016	31.7013
AL–HC-2	702.2451	30.4500
AL–HC-3	756.0441	32.7828
Average	729.7970 ± 26.9232	31.6447 ± 1.1674
AL–HG-1	650.9304	28.2250
AL–HG-2	720.3327	31.2343
AL–HG-3	828.9044	35.9421
Average	733.3892 ± 89.7025	31.8005 ± 3.8896

Note: CBS denotes curved beam strength; ILTS represents interlaminar tensile strength.

**Table A2:** Mechanical results of the four laminate types in the short beam test.

Specimens No.	Peak Force ± SD (N)	ILSS ± SD (MPa)
AL–CF-1	1853	43.4297
AL–CF-2	1713	40.1484
AL–CF-3	1740	40.8047
AL–CF-4	1838	43.0781
Average	1786.25 ± 69.8522	41.8652 ± 1.6321
AL–GF-1	1837	43.0547
AL–GF-2	1746	40.9219
AL–GF-3	1658	38.8593
AL–GF-4	1769	41.4609
Average	1752.5 ± 73.9031	41.0742 ± 1.7321
AL–HC-1	1804	42.2813
AL–HC-2	1806	42.3281
AL–HC-3	1747	40.9453
AL–HC-4	1754	41.1094
Average	1817.08 ± 31.6056	41.6660 ± 0.7408
AL–HG-1	1699	39.8203
AL–HG-2	1859	43.5703
AL–HG-3	1704	39.9375
AL–HG-4	1740	40.7813
Average	1750.5 ± 74.6034	41.0273 ± 1.7485

Note: ILSS represents interlaminar shear strength.

**Table A3:** Mechanical results of the four laminate types in the DCB test.

Specimens No.	$G_{Ic,init}$ ± SD (kJ/m^2^)	$G_{Ic,prop}$ ± SD (kJ/m^2^)
AL–CF-1	0.5629	0.7204
AL–CF-2	0.5098	0.5968
AL–CF-3	0.3639	0.4922
Average	0.4789 ± 0.1030	0.6031 ± 0.1142
AL–GF-1	0.4263	0.5175
AL–GF-2	0.5102	0.5780
AL–GF-3	0.3948	0.5268
Average	0.4438 ± 0.0597	0.5408 ± 0.0326
AL–HC-1	0.5523	0.7280
AL–HC-2	0.5024	0.6314
AL–HC-3	0.3748	0.5499
Average	0.4765 ± 0.0915	0.6364 ± 0.0892
AL–HG-1	0.6031	0.6426
AL–HG-2	0.5266	0.5674
AL–HG-3	0.5573	0.6259
Average	0.5623 ± 0.0385	0.6120 ± 0.0395

Note: $G_{Ic,\;init}$ denotes initiation toughness; $G_{Ic,\;prop}$ is propagation toughness.

**Table A4:** Mechanical results of the four laminate types in the ENF test.

Specimens No.	Intercept A	Slope m	$G_{IIc}$ ± SD (kJ/m^2^)
AL–CF-1	0.00722	1.00 × 10^−7^	0.7514
AL–CF-2	0.00738	1.06 × 10^−7^	0.6999
AL–CF-3	0.00754	7.32 × 10^−8^	0.6117
Average	0.00738	9.32 × 10^−8^	0.6877 ± 0.0707
AL–GF-1	0.01071	8.00 × 10^−8^	0.6239
AL–GF-2	0.01044	6.63 × 10^−8^	0.6996
AL–GF-3	0.01016	8.50 × 10^−8^	0.6808
Average	0.01044	7.71 × 10^−8^	0.6681 ± 0.0394
AL–HC-1	0.00757	9.28 × 10^−8^	0.7618
AL–HC-2	0.00625	8.21 × 10^−8^	0.8863
AL–HC-3	0.00779	1.00 × 10^−7^	0.7342
Average	0.00721	9.17 × 10^−8^	0.7941 ± 0.0810
AL–HG-1	0.01411	9.56 × 10^−8^	0.5679
AL–HG-2	0.00981	5.55 × 10^−8^	0.6025
AL–HG-3	0.00982	7.83 × 10^−8^	0.7775
Average	0.01125	7.65 × 10^−8^	0.6493 ± 0.1124

Note: A denotes the intercept of the compliance–crack-length calibration; m: represents the slope coefficient associated with the $a^{3}$ term; $G_{IIc}$ is the mode-II fracture toughness.

## References

[1] Li M., Wang Y., Niu Z., Yang S. (2020). Study on the weld-bonding process optimization and mechanical performance of aluminum alloy joints. Automot. Inno..

[2] Liu Q., Ou Z., Mo Z., Li Q., Qu D. (2015). Experimental investigation into dynamic axial impact responses of double hat shaped CFRP tubes. Compos. Part B Eng..

[3] Wang Z., Jin X., Li Q., Sun G. (2020). On crashworthiness design of hybrid metal-composite structures. Int. J. Mech. Sci..

[4] Dursun T., Soutis C. (2014). Recent developments in advanced aircraft aluminium alloys. Mater. Des. (1980–2015).

[5] Huang Z., Zhang X., Yang C. (2019). Static and dynamic axial crushing of Al/CRFP hybrid tubes with single-cell and multi-cell sections. Compos. Struct..

[6] Chen D., Luo Q., Meng M., Li Q., Sun G. (2019). Low velocity impact behavior of interlayer hybrid composite laminates with carbon/glass/basalt fibres. Compos. Part B Eng..

[7] Chang P.Y., Yeh P.C., Yang J.M. (2008). Fatigue crack initiation in hybrid boron/glass/aluminum fiber metal laminates. Mater. Sci. Eng. A-Struct. Mater. Prop. Microstruct. Process..

[8] Soltani P., Keikhosravy M., Oskouei R.H., Soutis C. (2011). Studying the tensile behaviour of GLARE laminates: A finite element modelling approach. Appl. Compos. Mater..

[9] Muniyan V., Kumar V.V., Suyambulingam I., Priyadharshini S., Divakaran D., Rangappa S.M., Siengchin S. (2025). A review of recent advancements in the impact response of fiber metal laminates. Heliyon.

[10] Morinière F.D., Alderliesten R.C., Sadighi M., Benedictus R. (2013). An integrated study on the low-velocity impact response of the GLARE fibre-metal laminate. Compos. Struct..

[11] Serubibi A., Hazell P.J., Escobedo J.P., Wang H., Oromiehie E., Prusty G.B., Phillips A.W., St John N.A. (2023). Fibre-metal laminate structures: High-velocity impact, penetration, and blast loading—A review. Compos. Part A Appl. Sci. Manuf..

[12] Vijayan M., Selladurai V., Vijay Kumar V., Balaganesan G., Marimuthu K. (2022). Low-velocity impact response of nano-silica reinforced aluminum/PU/GFRP laminates. Proceedings of the International Symposium on Plasticity and Impact Mechanics.

[13] Wang S., Liu M., Araby S., Wang X., Abdelsalam A.A., Xue H., Meng Q. (2023). Reinforcing interlaminar interface of carbon fiber reinforced metal laminates by graphene. Compos. Struct..

[14] Benzeggagh M.L., Kenane M.J.C.S. (1996). Measurement of mixed-mode delamination fracture toughness of unidirectional glass/epoxy composites with mixed-mode bending apparatus. Compos. Sci. Technol..

[15] Davidson B.D., Sediles F.O. (2011). Mixed-mode I–II–III delamination toughness determination via a shear–torsion-bending test. Compos. Part A Appl. Sci. Manuf..

[16] Turon A., Davila C.G., Camanho P.P., Costa J. (2007). An engineering solution for mesh size effects in the simulation of delamination using cohesive zone models. Eng. Fract. Mech..

[17] Camanho P.P., Davila C.G., de Moura M.F. (2003). Numerical simulation of mixed-mode progressive delamination in composite materials. J. Compos. Mater..

[18] Abdullah S.S., Bokti S.K., Wong K.J., Johar M., Chong W.W.F., Dong Y. (2024). Mode II and mode III delamination of carbon fiber/epoxy composite laminates subjected to a four-point bending mechanism. Compos. Part B Eng..

[19] Brunner A.J., Blackman B.R.K., Davies P. (2008). A status report on delamination resistance testing of polymer–matrix composites. Eng. Fract. Mech..

[20] Wilk J. (2019). Applicability of mode II interlaminar fracture toughness testing methods for characterization of thermoplastic laminates with woven fabric reinforcements. Eng. Fract. Mech..

[21] Lopes R.M., Campilho R.D.S.G., Da Silva F.J.G., Faneco T.M.S. (2016). Comparative evaluation of the Double-Cantilever Beam and Tapered Double-Cantilever Beam tests for estimation of the tensile fracture toughness of adhesive joints. Int. J. Adhes. Adhes..

[22] Ranz D., Cuartero J., Miravete A., Miralbes R. (2017). Experimental research into interlaminar tensile strength of carbon/epoxy laminated curved beams. Compos. Struct..

[23] Makeev A., Carpentier P., Shonkwiler B. (2014). Methods to measure interlaminar tensile modulus of composites. Compos. Part A Appl. Sci. Manuf..

[24] Fisher J., Czabaj M.W. (2024). A new test for characterization of interlaminar tensile strength of tape-laminate composites. Compos. Part A Appl. Sci. Manuf..

[25] Rosselli F., Santare M.H. (1997). Comparison of the short beam shear (SBS) and interlaminar shear device (ISD) tests. Compos. Part A Appl. Sci. Manuf..

[26] Selmy A.I., Elsesi A.R., Azab N.A., Abd El-baky M.A. (2012). Interlaminar shear behavior of unidirectional glass fiber (U)/random glass fiber (R)/epoxy hybrid and non-hybrid composite laminates. Compos. Part B Eng..

[27] Gagani A.I., Krauklis A.E., Sæter E., Vedvik N.P., Echtermeyer A.T. (2019). A novel method for testing and determining ILSS for marine and offshore composites. Compos. Struct..

[28] Škec L., Alfano G., Jelenić G. (2019). Complete analytical solutions for double cantilever beam specimens with bi-linear quasi-brittle and brittle interfaces. Int. J. Fract..

[29] Xu W., Guo Z.Z. (2018). A simple method for determining the mode I interlaminar fracture toughness of composite without measuring the growing crack length. Eng. Fract. Mech..

[30] De Moura M.F.S.F., Morais J.J.L., Dourado N. (2008). A new data reduction scheme for mode I wood fracture characterization using the double cantilever beam test. Eng. Fract. Mech..

[31] Gong Y., Chen X., Li W., Zhao L., Tao J., Zhang J., Hu N. (2021). Delamination in carbon fiber epoxy DCB laminates with different stacking sequences: R-curve behavior and bridging traction-separation relation. Compos. Struct..

[32] Blackman B.R.K., Brunner A.J., Williams J.G. (2006). Mode II fracture testing of composites: A new look at an old problem. Eng. Fract. Mech..

[33] Wang W.X., Nakata M., Takao Y., Matsubara T. (2009). Experimental investigation on test methods for mode II interlaminar fracture testing of carbon fiber reinforced composites. Compos. Part A Appl. Sci. Manuf..

[34] Gliszczynski A., Wiącek N. (2021). Experimental and numerical benchmark study of mode II interlaminar fracture toughness of unidirectional GFRP laminates under shear loading using the end-notched flexure (ENF) test. Compos. Struct..

[35] Greisel M., Jäger J., Moosburger-Will J., Sause M.G., Mueller W.M., Horn S. (2014). Influence of residual thermal stress in carbon fiber-reinforced thermoplastic composites on interfacial fracture toughness evaluated by cyclic single-fiber push-out tests. Compos. Part A Appl. Sci. Manuf..

[36] Yu Y., He K., Hang Z., Liu W., Zhao W. (2024). Effects of adhesive layer thickness on the fracture properties of a concrete-epoxy resin interface. Theor. Appl. Fract. Mech..

[37] Bonhin E.P., Botelho E.C., Ribeiro M.V. (2022). Interlaminar shear of FML produced with surface treatment by mechanical abrasion. Procedia CIRP.

[38] He M.Y., Evans A.G., Hutchinson J.W. (1994). Crack deflection at an interface between dissimilar elastic materials: Role of residual stresses. Int. J. Solids Struct..

[39] de Freitas S.T., Sinke J. (2015). Test method to assess interface adhesion in composite bonding. Appl. Adhe. Sci..

[40] Barbosa N.G.C., Campilho R.D.S.G., Silva F.J.G., Moreira R.D.F. (2018). Comparison of different adhesively-bonded joint types for mechanical structures. Appl. Adhes. Sci..

[41] Yu T., Fernando D., Teng J.G., Zhao X.L. (2012). Experimental study on CFRP-to-steel bonded interfaces. Compos. Part B Eng..

[42] Bennati S., Colleluori M., Corigliano D., Valvo P.S. (2009). An enhanced beam-theory model of the asymmetric double cantilever beam (ADCB) test for composite laminates. Compos. Sci. Technol..

[43] Wang W., De Freitas S.T., Poulis J.A., Zarouchas D. (2021). A review of experimental and theoretical fracture characterization of bi-material bonded joints. Compos. Part B Eng..

[44] Tsokanas P. (2023). Fracture Toughness of Metal-to-Composite Adhesive Joints with Bending–Extension Coupling and Residual Thermal Stresses. Fracture Analysis of Layered Beams with an Elastically Coupled Behavior and Hygrothermal Stresses: Application to Metal-to-Composite Adhesive Joints.

[45] Bakhbergen U., Abbassi F., Kalimuldina G., Montazami R., Shehab E., Araby S. (2024). Recent approaches of interface strengthening in fibre metal laminates: Processes, measurements, properties and numerical analysis. Compos. Part B Eng..

[46] Laban O., Mahdi E. (2017). Enhancing mode I inter-laminar fracture toughness of aluminum/fiberglass fiber-metal laminates by combining surface pre-treatments. Int. J. Adhes. Adhes..

[47] Hua X., Li H., Lu Y., Chen Y., Qiu L., Tao J. (2019). Interlaminar fracture toughness of GLARE laminates based on asymmetric double cantilever beam (ADCB). Compos. Part B Eng..

[48] Liu C., Du D., Li H., Hu Y., Xu Y., Tian J., Tao G., Tao J. (2016). Interlaminar failure behavior of GLARE laminates under short-beam three-point-bending load. Compos. Part B Eng..

[49] (2022). Standard Test Method for Measuring the Curved Beam Strength of a Fiber-Reinforced Polymer-Matrix Composite.

[50] (2022). Standard Test Method for Short-Beam Strength of Polymer Matrix Composite Materials and Their Laminates.

[51] (2021). Standard Test Method for Mode I Interlaminar Fracture Toughness of Unidirectional Fiber-Reinforced Polymer Matrix Composites.

[52] (2019). Standard Test Method for Determination of the Mode II Interlaminar Fracture Toughness of Unidirectional Fiber-Reinforced Polymer Matrix Composites.

